# Biomarkers for Sepsis

**DOI:** 10.1155/2014/547818

**Published:** 2014-03-30

**Authors:** Cesar Henriquez-Camacho, Juan Losa

**Affiliations:** Hospital Universitario Fundacion Alorcon, Calle Valdelaguna 1, 28922 Madrid, Spain

## Abstract

Bloodstream infections are a major concern because of high levels of antibiotic consumption and of the increasing prevalence of antimicrobial resistance. Bacteraemia is identified in a small percentage of patients with signs and symptoms of sepsis. Biomarkers are widely used in clinical practice and they are useful for monitoring the infectious process. Procalcitonin (PCT) and C-reactive protein (CRP) have been most widely used, but even these have limited abilities to distinguish sepsis from other inflammatory conditions or to predict outcome. PCT has been used to guide empirical antibacterial therapy in patients with respiratory infections and help to determine if antibacterial therapy can be stopped. New biomarkers such as those in this review will discuss the major types of biomarkers of bloodstream infections/sepsis, including soluble triggering receptor expressed on myeloid cells-1 (sTREM-1), soluble urokinase-type plasminogen receptor (suPAR), proadrenomedullin (ProADM), and presepsin.

## 1. Introduction

“Sepsis is a state caused by microbial invasion from a local infectious source into the bloodstream which leads to signs of systemic illness in remote organs,” this was the first scientific definition of sepsis proposed by Dr. Schottmuller in 1914 [1]. Thus, bloodstream infection or bacteremia was a condition to the diagnosis of sepsis and this definition did not change significantly over the years. Sepsis, septicemia, and bloodstream infections (bacteremia) were considered to refer to the same clinical condition, and, in practice, the terms were often used interchangeably. Now, we know that less than one-half of the patients who have signs and symptoms of sepsis have positive blood culture or other microbiological proof of an infectious focus [2].

Bloodstream infections are a major concern to physicians because of high levels of antibiotic consumption and of the increasing prevalence of antimicrobial resistance. Thus, they lack accuracy to tailor subsequent therapy.

Blood cultures to detect bloodstream infections are the mainstay of such attempts when patients do not display localizing signs or symptoms. The presence of SIRS has been shown to increase the likelihood that the blood culture will be positive but blood cultures are often negative in patients with clinical sepsis [3].

Bloodstream infections can produce an immune response to bacterial endotoxins. Innate immune response stimulates macrophages to produce tumor necrosis factor (TNF), interleukin-1*β*, and interleukin-6. These three proinflammatory cytokines produce a systemic inflammatory response syndrome (SIRS) which is characteristic of early sepsis. A compensatory anti-inflammatory response syndrome (CARS) has been described by Bone [4] that often follows the hyperinflammatory phase, especially in patients who develop what is called “severe” sepsis. In severe sepsis, evidence of widespread organ dysfunction is also present, including multiorgan dysfunction (lung, liver, and/or kidney injury). The so-called septic shock, in which patients suffer cardiovascular collapse unresponsive to fluid resuscitation and vasopressor therapy, is often the terminal event of severe sepsis [5].

However, no gold standard exists for proof of infection. Bacteremia is identified in only about 30% of patients with sepsis, depending on previous antibiotic treatment [6].

Biomarkers can add accuracy of any bacterial presence and they are useful to monitoring the evolution of infectious process. New biomarkers related to infectious diseases have been tested the last years but few of them, however, have gone through the hurdles of rigorous testing to be used in the clinical practice [7].

Several biomarkers are already available for clinical use in sepsis; however, their effectiveness in many instances is limited by the lack of specificity and sensitivity. Other factors include limitation to characterize the presence of an infection and the complexity of the inflammatory and immune processes to stratify patients into homogenous groups for specific treatments [8].

Many biomarkers can be used in sepsis, but none has sufficient specificity or sensitivity to be routinely employed in clinical practice. PCT and CRP have been most widely used, but even these have limited abilities to distinguish sepsis from other inflammatory conditions or to predict outcome. In view of the complexity of the sepsis response, it is unlikely that a single ideal biomarker will ever be found [9].

In the 1980s, there were numerous studies about the C-reactive protein (CRP), a well-established member of the group of proteins synthesised in the liver. In the 1990s, investigators discovered that the levels of procalcitonin (PCT), the precursor of the hormone calcitonin, were elevated in patients with bacterial infection [10]. Elevations of both CRP and PCT were added to the updated definition of sepsis in 2003. Then, in the early part of the past decade, clinical guides of intensive “goal-directed” treatment of severe sepsis and septic shock used elevated lactate levels to guide therapy, and obtaining a lactate level when monitoring patients at risk of developing sepsis became standard practice [11].

No single biomarker of bloodstream infections may be ideal, but many are helpful in terms of identifying bacterial infections in critically ill patients who need close monitoring so that the antibiotic therapy may be modified or stopped as soon as possible. This review will discuss the major types of biomarkers of bloodstream infections/sepsis which have been tested in different conditions.

## 2. CRP

CRP is a protein produced in response to infection and/or inflammation and it is widely used in clinical tests to diagnose and manage patients with sepsis. This biomarker is an acute phase reactant whose synthesis in the liver is upregulated by IL-6. The CRP's role during acute inflammation is not entirely clear and it may bind the phospholipid components of microorganisms, facilitating their removal by macrophages. Because the levels of CRP rise significantly during acute inflammation, this biomarker has been used for decades to indicate the presence of significant inflammatory or infectious disease, especially in pediatrics [12]. Although its low specificity may be its primary drawback as a biomarker of sepsis in adults, it is commonly used to screen for early onset sepsis in neonatology [13].

## 3. Procalcitonin

Procalcitonin is a prohormone (peptide precursor) of calcitonin that is released by parenchymal cells, including liver cells, kidney cells, adipocytes, and muscle cells in response to bacterial toxins, leading to elevated serum levels (up to 5000-fold) within 2 to 4 hours; in contrast, procalcitonin is downregulated in patients with viral infections [14]. The biological half-life of PCT is 22 to 26 hours, an advantageous time point compared with CRP and other acute-phase reactants [15].

Although elevations of PCT can be observed in noninfectious disorders, especially following trauma [16], at present, PCT levels have been used to guide empirical antibacterial therapy in patients with acute exacerbations of chronic bronchitis, community-acquired pneumonia (CAP), and sepsis. Also, PCT levels, along with standard clinical parameters, can assist in determining whether the patient's empirical antibacterial therapy is effective [17]. Higher PCT levels have been associated with increased mortality rates and correlated with severity scores (APACHE, SOFA, and SAPS) [18]. Finally, the most useful application is the use of sequential PCT levels to determine if antibacterial therapy can be stopped [19].

### 3.1. Procalcitonin for the Guidance of Antibiotic Therapy in Lower Respiratory Tract Infections

Numerous studies have evaluated PCT as a biomarker to guide initiation of antibiotic therapy in patients suspected of lower respiratory tract infections. A meta-analysis published in 2011 with 8 studies (3431 patients) showed a reduction in antibiotic prescription in the PCT-guided antibiotic treatment groups with a RR: 0.69 (CI 95%: 0.55 to 0.88) but with a significant heterogeneity (*χ* ^2^ = 192.34; *P* < 0.001, *I* ^2^ = 96.9%) [20]. As PCT levels increase upon bacterial infection and decrease upon recovery, it can be used to guide antibiotic therapy in individual patients as a surrogate biomarker. Two low PCT measurements, over the first 4 to 6 hours of hospital admission, resulted in fewer patients started on empirical antibacterials. Low PCT levels over the first 4 hours of inpatient care have an excellent negative predictive value for bacterial infection [21].

A Cochrane review published in 2012 with 14 studies (4221 participants) showed that PCT guidance was not associated with increased mortality (5.7% versus 6.3%, adjusted OR 0.94, 95% CI 0.71 to 1.23) or treatment failure (19.1% versus 21.9%). Total antibiotic exposure was significantly reduced overall [21]. Similar results were founded in a recent meta-analysis including 7 studies (1075 patients) with a hazard ratio of 1.27, 95% CI: 1.01–1.53 reduction of antimicrobial therapy [22].

To date, numerous studies (including meta-analysis) have been published and provide consistent results that withhold antibiotic prescription can be done with low levels of PCT (<0.25 ng/mL) [23].

### 3.2. Procalcitonin for Antibiotic Guidance in Other Infections

Procalcitonin has been studied in febrile neutropenic patients, fungal infections, postoperative fever, arthritis, endocarditis, meningitis, and suspected bloodstream infections [24–26]. The majority of published studies were observational and it remains uncertain whether PCT can be safely used for antibiotic guidance in different settings. For some infections, PCT may not be sensitive enough for routine clinical use. In a recent meta-analysis with 6 trials (1006 episodes of suspected endocarditis), the global measures of accuracy of CRP were higher than PCT showing that current evidence does not support the routine use of serum PCT or CRP to rule in or rule out endocarditis [27].

### 3.3. Procalcitonin for Identification of Sepsis

Procalcitonin has been studied to differentiate between sepsis and systemic inflammatory response syndrome of noninfectious origin. Numerous studies have investigated the diagnostic usefulness of PCT, comparing it with CRP. Initially, PCT was found more sensitive and specific than CRP for bacterial infection [28].

In a meta-analysis of Uzzan et al. (publication date: 2006), 33 studies published between April, 1996, and October, 2004, were included, with 3,943 patients (1,825 patients with sepsis, severe sepsis, or septic shock and 1,545 with only systemic inflammatory response syndrome). This meta-analysis showed that the summary receiver operating characteristics curve for PCT was higher than for CRP for identification of sepsis (0.78 versus 0.71, *P* = 0.02). However, the investigators restricted the population to surgery or trauma patients. Therefore, no conclusion can be drawn for patients other than surgical [29].

A posterior meta-analysis (2007) looking at the diagnostic accuracy of PCT in sepsis diagnosis in critically ill patients included 18 studies published between April, 1996, and November, 2005, with very restrictive inclusion criteria, including evidence of infection by any microbiological test. Uzzan et al. concluded that PCT was not able to discriminate between sepsis and systemic inflammatory response syndrome. The diagnostic accuracy of PCT was low, mean sensitivity and specificity were both 71% (95% CI 67–76), and the area under the summary receiver operator characteristic curve was 0.78 (95% CI 0.73–83). However, their findings were heavily biased because of their selection criteria. The rejection of such studies has been raised as a major criticism of their conclusion that PCT cannot accurately distinguish sepsis from SIRS in critically ill patients [30].

The most recent meta-analysis published by Tang et al. included 30 studies (3244 patients) until February 2012. They concluded that accuracy of PCT to discriminate sepsis and systemic inflammatory response was low, mean sensitivity 77% (95% 72–81), and specificity 79% (95% CI 74–84). The area under the receiver operating characteristic curve was 0.85 (95% CI 0.81–0.88), with substantial heterogeneity (*I* ^2^: 96%, 95% CI 94–99) [31].

Although PCT has been shown to correlate closely with infection, it has some limitations. It rises transiently in patients with nonseptic conditions and systemic inflammatory response syndromes (SIRS) (e.g., trauma, surgery, and heatstroke) and is not detectable in certain cases of sepsis [32].

## 4. New Biomarkers

There are new biomarkers tested for acute infections with different diagnostic and prognostic value (see Table 1). In adults, the soluble triggering receptor expressed on myeloid cells-1 (sTREM-1), soluble urokinase-type plasminogen receptor (suPAR), proadrenomedullin (pro-ADM), and presepsin appear promising because of acceptable sensitivity and specificity [7] (see Table 2).

### 4.1. sTREM-1

The triggering receptor expressed on myeloid cells-1 (TREM-1) is a member of the immunoglobulin superfamily. Its expression on phagocytes is upregulated by exposure to bacteria and fungi. A soluble form of TREM-1 (sTREM-1) can be found in body fluids, such as plasma, pleural fluid, bronchoalveolar lavage fluid, urine, and cerebrospinal fluid, where it can be assayed by ELISA using commercial immunoassay kits [33].

Clinical studies of the ability of the soluble form of TREM-1 to reliably identify patients with sepsis have not been promising [34]. However a meta-analysis of 11 studies (1795 patients included) showed a pooled sensitivity and specificity of 79% (95% confidence interval (CI), 65 to 89) and 80% (95% CI, 69 to 88), respectively with ROC curve of 0.87 (95% CI, 0.84 to 0.89). In this meta-analysis, for a prevalence of 62% of sepsis, the negative predictive value (NPV) was 0.7 and the positive predictive value (PPV) is 0.86. Finally, plasma sTREM-1 had a moderate diagnostic performance in differentiating sepsis from SIRS and was not sufficient for sepsis diagnosis in systemic inflammatory patients [35].

### 4.2. suPAR

The soluble form of urokinase-type plasminogen activator receptor (suPAR) is a new biological marker of immunologic activation [36]. Urokinase-type plasminogen activator receptor (uPAR) is expressed on various cell types and participates in numerous immunologic functions including migration, adhesion, angiogenesis, fibrinolysis, and cell proliferation. uPAR/uPA system participated in migration of inflammatory cells from the bloodstream into tissues against infection. During inflammatory stimulation, uPAR is cleaved from the cell surface by proteases to create the soluble form of the receptor, suPAR, which can be detected in blood, urine, and cerebrospinal fluid [37]. Measurements can be obtained from commercial ELISA kits; suPAR measurements also are included in multiplex assays together with cytokines.

High serum suPAR concentrations have also been found to predict mortality in patients with active tuberculosis and other diseases associated with an inflammatory response [38].

Some studies have showed that suPAR levels were elevated in acutely ill patients but that their diagnostic value was not superior to other biomarkers such as CRP, PCT, or sTREM-1 [39]. Recently, two studies evaluating diagnostic accuracy of suPAR have shown specificity from 64–77% [40, 41].

### 4.3. Pro-ADM

Adrenomedullin (ADM) is a 52-amino-acid peptide with immune modulating, metabolic, and vasodilator activity. Its widespread production in the tissues helps to maintain a blood supply in every organ. Moreover, ADM has a bactericidal activity and could be helpful in the evaluation of sepsis diagnosis and prognosis and in monitoring such conditions [42]. Prohormone fragments (pro-ADM) are more stable than the complete peptide and their levels can be measured in biological fluids by automated methods using the TRACE (Time-Resolved Amplified Cryptate Emission) method after immunocapture. The midregional fragment of proadrenomedullin (MR-pro-ADM), included between amino acids 45–92, is the most stable part of the ADM, and it has been detected in plasma of patients with septic shock as a consequence of the ADM active peptide degradation [43].

Pro-ADM is a biomarker of prognostic value and could be used to identify more severe patients with pneumonia and/or needing ICU care [44].

In a recent single prospective observational study conducted in a Spanish adult intensive care unit (137 patients), pro-ADM showed a significant dose-response trends to predict hospital mortality (OR = 3.00, 95% CI 1.06–8.46) compared to PCT and CRP. However, the prognostic accuracy was better for severity scores than for any biomarker [45].

In an Italian study comparing PCT and MR-pro-ADM in 200 septic patients, 90 patients with SIRS, and 30 healthy individuals, the pro-ADM distinguished septic patients. Moreover, the combined use of PCT and MR-pro-ADM gave a posttest probability of 0.998 in the cohort of all septic patients. The combination of biomarkers may substantially improve the early diagnosis of sepsis [46].

### 4.4. Presepsin

Cluster of differentiation 14 (CD14) is a glycoprotein expressed on the membrane surface of monocytes and macrophages and serves as a receptor for lipopolysaccharides (LPSs) and LPS-binding proteins (LPBs). By activating a proinflammatory signaling cascade on contact with infectious agents, CD14 has a role as a recognition molecule in the innate immune response against microorganisms. During inflammation, plasma protease activity generates soluble CD14 (sCD14) fragments. One of them, called sCD14 subtype (sCD14-ST), or presepsin, is normally present in very low concentrations in the serum of healthy individuals and has been shown to be increased in response to bacterial infections [47]. Plasma levels of presepsin can be measured using an automated chemoluminescent assay (PATHFAST).

In a multicenter prospective study (106 patients with suspected sepsis or septic shock were included and 83 SIRS patients without infection), elevated concentrations of presepsin were observed in septic patients compared to control patients [48]. The best diagnostic cutoff for presepsin was 600 pg/mL with sensitivity of 78.95% (95% CI, 69.4 to 86.6) and specificity of 61.90% (95% CI, 50.7 to 72.3). There was no difference between levels of presepsin and sepsis severity. Moreover, the area under the curve (AUC) calculated for PCT was wider, demonstrating a better diagnostic accuracy than presepsin. Although presepsin showed a significant prognostic value and initial values were significantly correlated with in-hospital mortality of patients affected by sepsis, severe sepsis, or septic shock, two recent studies have shown that presepsin is an useful biomarker for early diagnosis of sepsis and evaluation of prognosis in septic patients (sensitivity: 71-72%, specificity: 70–86%, and NPV: 52–71%) [49, 50].

## 5. Conclusions

1. Bloodstream infection is a serious life-threatening condition with high mortality. In some cases, the diagnosis is challenging. An early diagnosis of sepsis helps to enable rapid treatment, improve outcomes, and reduce unnecessary antibiotic therapy.
2. Choosing the correct empiric therapy is sometimes a difficult process. The emergence of resistant pathogens is consequence of irrational use of antibiotics.
3. PCT and PCR are widely used in clinical practice and are more useful to rule out infection. PCT is the most studied biomarker that guides early stopping of antibiotic therapy in adults.
4. New biomarkers are being evaluated in different clinical scenarios, although none of them have shown sufficient sensitivity or specificity to rule out infection.
5. Presepsin appears to be the most promising new biomarker for early diagnosis of sepsis and better prognostic performance than procalcitonin.

## Figures and Tables

**Table 1:** Role of biomarkers of sepsis.

Biomarkers of sepsis	Prognostic value	Diagnostic value	Syndrome/disease
CRP	No	Yes	Sepsis
Procalcitonin	Yes	Yes	Sepsis/respiratory tract infections/pneumonia/
sTREM-1	Yes	Yes	Sepsis/pneumonia/ meningitis
Pro-ADM	Yes	No	Pneumonia
suPAR	Yes	No	Sepsis/tuberculosis
Presepsin	Yes	Yes	SIRS/sepsis

**Table 2:** Evaluation of new biomarkers of sepsis.

New Biomarkers	Level	Sensit.	Specif.	AUC	NPV	PPV	Prevalence (%)	Type	Study
sTREM-1 (pg/mL)	40–755*	79	80	0.87	70	86	1113/1795 (62)	Diagnostic	[34]

Pro-ADM (nmol/L)	4.86	53	84	0.72	77	64	47/137 (34.7)	Prognostic	[44]

suPAR (ng/mL)	10	80	77	0.79	95	42	27/125 (21.6)	Diagnostic	[39]
	8.9	66	64	0.73	76	50	94/258 (36.43)	Diagnostic	[40]

Presepsin (pg/mL)	2866	79	62	0.70	87	45	55/189 (29)	Diagnostic	[47]
	1606	72	70	0.74	71	71	71/100 (71)	Prognostic	[48]
	317	71	86	0.82	52	93	372/859 (43.3)	Diagnostic	[49]
	556	62	67	nr	78	48	283/859 (32.94)	Prognostic	[49]

Sensit.: sensitivity, specif: specificity, AUC: area under curve, NPV: negative predictive value, PPV: positive predictive value, and nr: not reported.

*Cutoff point based in a meta-analysis of 11 studies.
